# HLA alleles associated with COVID-19 susceptibility and severity in different populations: a systematic review

**DOI:** 10.1186/s43042-023-00390-5

**Published:** 2023-01-22

**Authors:** Meryem Fakhkhari, Hayat Caidi, Khalid Sadki

**Affiliations:** 1 Research Laboratory in Oral Biology and Biotechnology, Faculty of Dental Medicine, Mohammed V University in Rabat, Rabat, Morocco; 2 NARST Surveillance Unit, National Center for Emerging and Zoonotic Infectious Diseases, Centers for Disease Control and Prevention, Atlanta, GA USA

**Keywords:** Association, HLA alleles, COVID-19 severity, COVID-19 susceptibility

## Abstract

**Background:**

COVID-19 is a respiratory disease caused by a novel coronavirus called as Severe Acute Respiratory Syndrome Coronavirus 2 (SARS-CoV-2). Detected for the first time in December 2019 in Wuhan and it has quickly spread all over the world in a couple of months and becoming a world pandemic. Symptoms of the disease and clinical outcomes are very different in infected people. These differences highlight the paramount need to study and understand the human genetic variation that occurring viral infections. Human leukocyte antigen (HLA) is an important component of the viral antigen presentation pathway, and it plays an essential role in conferring differential viral susceptibility and severity of diseases. HLA alleles have been involved in the immune response to viral diseases such as SARS-CoV-2.

**Main body of the abstract:**

Herein, we sought to evaluate this hypothesis by summarizing the association between HLA class I and class II alleles with COVID-19 susceptibility and/or severity reported in previous studies among different populations (Chinese, Italian, Iranian, Japanese, Spanish, etc.). The findings of all selected articles showed that several alleles have been found associated with COVID-19 susceptibility and severity. Even results across articles have been inconsistent and, in some cases, conflicting, highlighting that the association between the HLA system and the COVID‐19 outcome might be ethnic‐dependent, there were some alleles in common between some populations such as HLA-DRB1*15 and HLA-A*30:02.

**Conclusion:**

These contradictory findings warrant further large, and reproducible studies to decipher any possible genetic predisposition underlying susceptibility to SARS-COV-2 and disease progression and host immune response.

## Introduction

December 2019 marked the highly transmissible pandemic that caused considerable morbidity and mortality and drastically changed people’s everyday life. The pandemic caused by the novel coronavirus, SARS-COV-2, began in Wuhan, Hubei Province in China. It rapidly spread to Europe, the United States (US), and the rest of the world, manifesting as coronavirus disease 2019 (COVID-19) [1]. Despite being detected approximately three years ago, the number of confirmed cases and deaths related to it has continued to rise and the severity and fatality rate of the virus vary from country to another. The wide spectrum of clinical symptoms described among COVID-19 patients within different populations, which ranges from asymptomatic or mildly symptomatic infections to severe pneumonia, respiratory failure, multi-organ failure and death, is one of its characteristics that continues to surprise us [2, 3]. Furthermore, advanced age, comorbidities such as hypertension, cardiovascular disease, diabetes and also obesity have been identified as clinical risk factors for severe COVID-19 disease [1].

Although differences in infection susceptibility and severity between populations are difficult to explain, genetic and environmental factors play a crucial role in virus pathogenesis. A variety of research are being conducted on human genetic elements that may contribute to the noticed diversified disease severity, to better perceive how the clinical course may be influenced by patient’s genetics and to identify which aspects could be related to SARS-COV-2 clinical variability [4]. One of the genetic elements that may be responsible for the variations in virus susceptibility and severity is the polymorphism in the HLA system. This latter, is widely used as a strategy in the investigation for the etiology of infectious diseases and autoimmune disorders [4], and have been suggested as potential genetic host factors that affect individual immune response to SARS-CoV-2. HLA genetic variants have been shown to influence the clinical course of patients infected with several RNA viruses such as influenza virus H1N1, Hantavirus, SARS-CoV-1 and many other viruses [1, 5].

An individual’s ability to mount an effective immune response to viral infection is completely reliant upon their inherited complement of human leukocyte antigens that controls our adaptive immunity by presenting pathogenic peptides to CD8+ T lymphocytes through class I HLA and to CD4+ T lymphocytes through class II HLA as opposed to the case for many bacterial and parasitic infections. The HLA type of the patient is likely to be a major influencing factor in an infected individual’s ability to give rise to an effective immune response [6, 7].

Individual HLA alleles can influence viral infections susceptibility and severity. Within the case of COVID-19, such an analysis may additionally make a contribution to characterizing individuals at higher risk of the disease as well as understanding disparities among countries within the epidemic patterns at the epidemiological level [5].

Numerous research have looked into potential associations between the genetic variability in major histocompatibility complex (MHC) class I and Class II genes and the susceptibility to SARS-CoV-2 and severity of COVID-19. Herein, we summarize the association between Major Histocompatibility Complex and COVID-19 susceptibility and severity in different populations (refer to Table 1), in order to identify HLA alleles underlying the different rates of COVID-19 infection among patients and populations.

**Table 1:** Basic characteristics of the included articles

Typed Loci	Population	Sample size (cases/controls)		Study	Year
HLA-A/B/C, DRB1/3/4/5, DQA1, DQB1, DPA1, DPB1,	Chinese	82	3790	Wei Wang et al.	2020
HLA-A/B/C, DQA1, DPA1, DRB1/3/4/5, DQB1, DPB1	Italian	99	1017	Antonio Novelli et al.	2020
HLA-A, B, C/DRB1 and DQB1,	Spanish	72	3886	Lorente et al.	2020
33 different HLA-B alleles	Chinese	190	3829	Yung Yuk Lin et al.	2020
HLA-A, B, and C	Italian	NA	490,926	Pierpaolo Correale et al.	2020
HLA-A, B, C and DRB1	Italian	182	619	Roberto Littera et al.	2020
HLA class I(A, B,C) and II(DRB1, DQA1, DQB1, DPB1)	Chinese	332	NA*	Fang Wang et al.	2020
HLA-A, -B, -C, -DRB1/3/4/5, and HLA-DQB1	British	80	10,308	Poulton Kay et al.	2020
HLA-A, B, C/DRB1/ and DQB1	Saudi Arabian	135	135	Fatmah Naemi et al.	2021
HLA-A, -C, -B, -DQA1 and -DPA1/ (HLA-DRB1, -DRB3, -DRB4, -DRB5, -DQB1 and -DPB1)	Japanese	190	423	Seik-Soon Khor et al.	2021
HLA-DRB1 and DQB1	Iranian	144	153	Samaneh Ebrahimi et al.	2021
HLA-A, B and DR	Iranian	48	500	Ramin Hamidi Farahani et al.	2021
HLA-A, -B, -C, -DPA1, -DPB1, -DQA1, -DQB1, -DRB1, -DRB3/4/5	Midwestern United States	234	22,000	Emily Schindler et al.	2021
HLA class I (A, B and C) and class II (DRB1)	Japanese	178	NA*	Alitzel Anzurez et al.	2021
HLA-A, B, and DRB1	Italian	265	56,039	Antonio Amoroso et al.	2021
HLA-A, B, C and DRB1, DQA1, and DQB1	South Asian (Bangladeshis, Indians, and Pakistanis)	95	NA*	Fatmah Naemi et al.	2021
HLA-A, -B, -C, -DRB1, and -DQB1	Israelis	6413	66,499	Shay Ben Shachar et al.	2021
HLA class I (HLA-A, -B and -C) and II (HLA-DRB1 and HLA-DQB1)	Spanish	450	495	Juan Francisco Gutiérrez-Bautista et al.	2021

*NA, not available

## Research methods

### Search strategy

The following protocol was used to conduct the literature search for this review:

*Patients*: COVID-19 positive,

*Comparison*: HLA alleles,

*Outcomes*: Susceptibility, severity of COVID-19 in different populations,

### Literature search

The articles included in this review were found through a thorough search of related studies with the terms, Human Leukocyte Antigen association, COVID-19 or SARS-CoV-2 or COVID-19 Susceptibility. The aforementioned terms were combined with Boolean expression adapted to the scientific databases used in this review, which are: PubMed, Elsevier, Scopus, Web of Science, SpringerLink and Google scholar. These databases were chosen for their reputation of covering the most important and highest impact full-text journal’s paper and conference proceeding treating the fields treated in the current research.

### Study characteristics

We scoped for the association between SARS-CoV2 infection and the most susceptible HLA alleles worldwide.

A total of 46 articles were selected. Six papers were excluded after reading the title and abstract, and the full texts of the remaining 40 articles were reviewed. Finally, this review comprised 18 articles of HLA class I and class II alleles.

### Inclusion and exclusion criteria

**Fig. 1:**
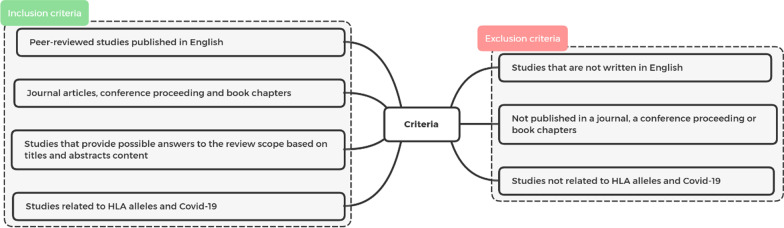
Inclusion and exclusion criteria

The selection of articles included in this review based on inclusion and exclusion criteria presented in Fig. 1.

### Data extraction

The following information were extracted from the selected articles: types of HLA Loci, population, number of cases and controls, author’s name and year of publication. The characteristics of selected articles are summarized in Table 1.

## Results and discussion

Multiple infectious diseases’ susceptibility and outcomes have been linked to the host’s genetic background in previous studies. In the human genome, HLA genes exhibit a high degree of polymorphism, making them essential for an effective antigen presentation to CD4+ and CD8+ T cells by Class I and Class II HLA, respectively, and for mounting appropriate immune responses to infectious agents. Human Leukocyte Antigen system encompasses a wide range of genes that play a critical role in the regulation and functioning of the immune system in combating viral infections [7–9].

Numerous studies have found that genetic variations at the loci encoding HLA genes can be associated to an increased or decreased risk of contracting infectious diseases. Research has uncovered a relationship between HLA alleles or haplotypes in major infectious diseases such as bacterial infections (tuberculosis, leprosy, melioidosis, and Staphylococcus aureus) and viral infections (human immunodeficiency virus (HIV) infection, hepatitis B, and hepatitis C) among several populations [7, 10]. It is speculative that various HLA alleles may determine the genetic susceptibility to or possibly protection against COVID-19 and can be related with severity, prognosis and disease outcomes [5].

Genotyping research on MHC class I and class II in COVID-19 cases have been done among different populations and its role in susceptibility or protection has been investigated. In this review, we sought to find HLA alleles associated with disease’s susceptibility and severity in different populations.

**Fig. 2:**
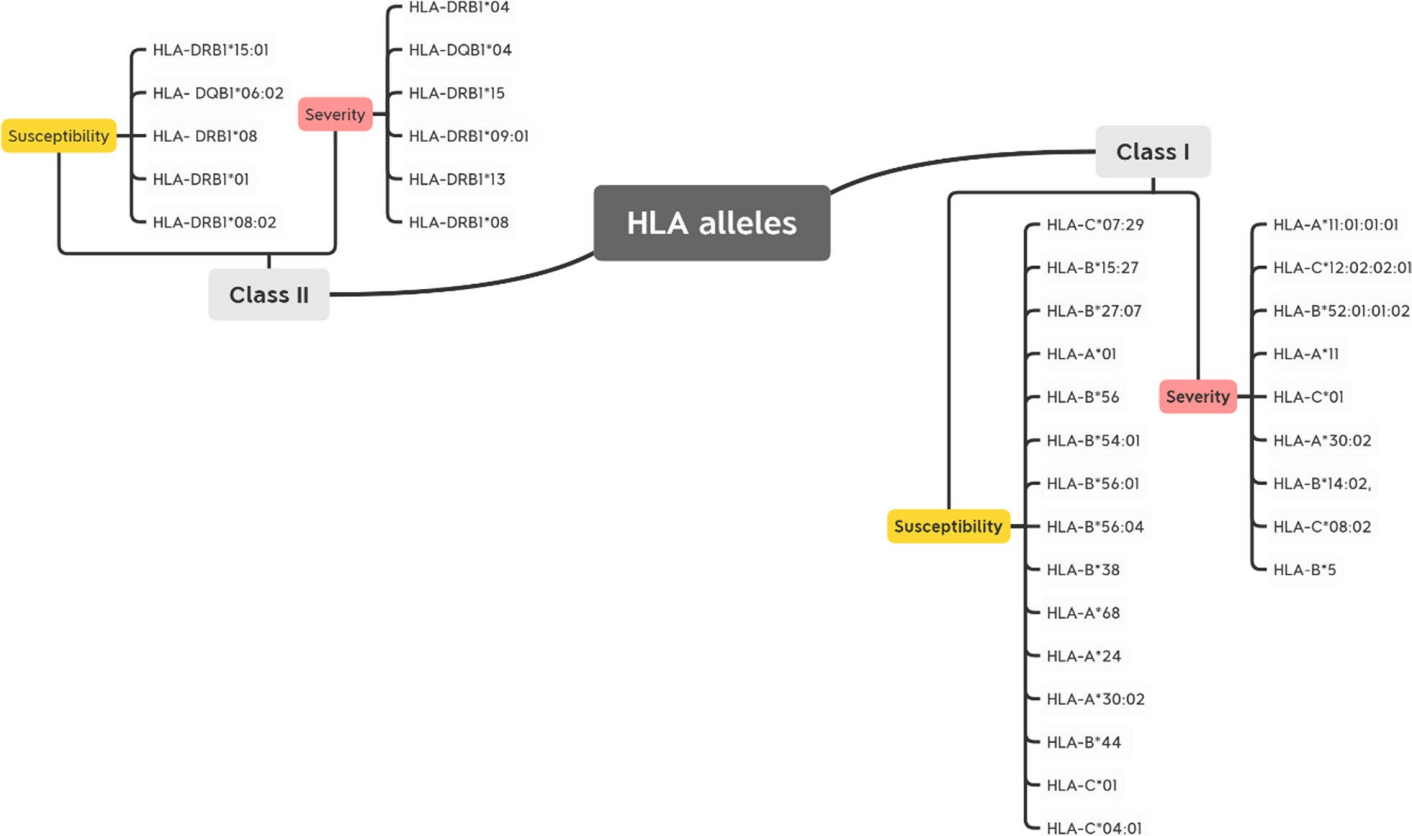
An overview of related HLA class I and class II alleles to COVID-19 susceptibility and severity in this review

A summary of the related HLA alleles to COVID-19 susceptibility or severity discussed in detail below is presented in Fig. 2.

The analysis of the 18 articles revealed correlations between several HLA alleles and susceptibility and severity of the disease among patients from different ethnicities. In Chinese population, two different set of HLA alleles were found more frequent in patients that in controls groups, HLA-C*07:29 & HLA-B*15:27 and HLA-B*54:01 & HLA-B*56:01 & HLA-B*56:04 as reported respectively by Wei Wang et al., and Yung et al., [11, 12]. Interestingly, this second set of HLA-B alleles belong to B22 serotype, suggesting the presence of one or more shared common residues that may be involved in COVID-19 disease susceptibility. Furthermore, it has been noted that five B22 alleles (HLA-B*54:01, HLA-B*55:01, HLA-B*55:07, HLA-B*55:12, and HLA-B*56:01) were found out of the 94 weakest HLA-B binders to SARS-CoV-2 [13], underlying the potential involvement of some B22 serotypes as a susceptibility marker. Moreover, in Italian population, other HLA alleles were observed to be significantly associated to COVID-19 severe form, HLA‐B*27, DRB1*15:01, and HLA‐DQB1*06:02 [14]. The results showed a substantial association between susceptibility to the disease and the outcomes.

Similarly, Poulton et al., Reported a significant positive association between HLA-DQB1*06 and HLA-DRB1*15:01 and COVID-19 disease in UK population. The study showed an impaired presentation of viral epitopes in those COVID-19 patients carrying HLA-DRB1*15:01, HLA-DQB1*06:02, HLA-DRB1*10, and HLA-A*26 alleles [6]. This, in turn, may prompt an inefficient T cell response and consequently inadequate antibody response, which is required for viral neutralization and elimination in synergy with T cell-mediated immunity. These findings are consistent with those previously published by Kachuri et al., and other studies, who identified DRB1 and DQB1 as significant genetic determinants influencing several diseases and especially viral infections susceptibilities such as for Epstein Barr virus (EBV), Varicella zoster virus (VZV), human herpesvirus 7, (HHV7), and Merkel cell polyomavirus (MCV) [11, 15–17].

The association between COVID-19 disease and HLA-DRB1*15 allele was also observed in Iranian population and indeed the allele frequency of HLA-DRB1*15 was found higher in patients with lymphopenia compared to those cases without lymphopenia. It has been reported that this clinical outcome was likely linked to the development of severe and critical COVID-19 disease, suggesting HLA-DRB1*15 allele as a genetic marker for severe form of the disease [18]. AL contrary, this finding is not replicated in another Iranian population but the authors reported a significant association between HLA-B*38, HLA-A*68, HLA-A*24, and HLA-DRB1*01 and the disease [19]. This lack of consistency between the Iranian studies could be due to the effect of the smaller samples size. It could be also explained by the implication of other factors such as age, sex, and environmental factors [20, 21].

In another Italy population study, seven HLA haplotypes were shown to be protective against SARS-CoV-2 infection (HLA-A*02:05-HLA-B*58:01) (HLA-A*02:05-HLA-C*07:01) (HLA-A*02:05-HLA-B*58:01-HLA-C*07:01) (HLA-A*02:05-HLA-B*18:01-HLA-DRB1*16:01) (HLA-A*02:05-HLA-B*58:01- HLA-DRB1*03:01) (HLA-A*02:05-HLA-B*58:01-HLA-C*07:01-DRB1*03:01) (HLA-A*02:05-HLA-B*58:01-HLA-C*07:01-HLA-DRB1*16:01 while five other haplotypes were found associated with an enhanced susceptibility to SARS-CoV-2 infection, (HLA-C*04:01-HLA-B*40:01-HLA-B*40:02) (HLA-A30:02-HLA-B*14:02) and (HLA-A30:02-HLA-C*08:02). After adjustment of the P-values, only the three-loci haplotype; HLA-A*30:02, HLA-B*14:02, HLA-C*08:02 maintained a statistically significant relation. This haplotype was shown to be highly associated with disease severity [22]. A previous study found that the hypersensitive reaction to nevirapine in Sardinian HIV-infected patients was associated with the (HLA-B*14:02-HLA-C*08:02) haplotype [23]. Besides, HLA-A*30:02 is part of an extended haplotype commonly found in Sardinia (HLA-A*30:02, B*18:01, C*05:01, DRB1*03:01) that is associated with autoimmune diseases such as multiple sclerosis and autoimmune type I hepatitis [22].

It has been reported that younger African American individuals carrying HLA-A*30:02 may be more susceptible to COVID-19 compared to other multiple population in the Midwest of the United States tested [24]. The majority of the HLA-A*30:02-carrying population are located in Africa. Referring to the Allele Frequency Net Database, HLA-A*30:02 which belongs to the A*30 serotypes, occurs in Lusaka, Zambia with the highest frequency of 23,3%, followed by Zimbabwe Harare Shona with a frequency of 14,7%, Senegal (12,4%), Cap Verde Northwestern Islands (12,1%) and, then South African Black with a frequency of 11,27%. In Lusaka, Zambia, COVID-19 positive deaths occurred in all age groups and it has been declared that COVID-19 was the leading cause of death during the peak of waves caused by the Beta and Delta variants, around 90% of deceased people were positive for SARS-COV-2 [25]. However, outcomes have been less severe among African populations and COVID-19 has been less deadly in Africa compared to other continents. It is strongly recommended that further studies evaluate the association of HLA-A*30:02 to COVID-19 severity in the African region.

Moreover, another Italian study examined the hypothesis that the large discrepancies observed in SARS-CoV-2 transmission between the North and the South of Italy may be explained by the regional prevalence of specific HLA class I alleles. They showed that HLA-A*25, HLA-B*08, HLA-B*44, HLA-B*15:01, HLA-B*51, HLA-C*01, and HLA-C*03 were positively associated with COVID-19 incidence, whereas HLA-B*14, HLA-B*18, and HLA-B*49 were found to be associated to the protection against SARS-CoV-2 infection. After employing a multiple regression model to minimize confounding factors, HLA-C*01 and HLA-B*44 alleles, which are more prevalent in northern Italy, remained positively associated with COVID-19 incidence. This epidemiologic analysis identified putative permissive class I alleles that are potentially unfit to trigger an efficient immune response capable to counteract SARS-CoV-2 infection. Both HLA-B*44 and HLA-C*01 alleles, which have been associated with known inflammatory autoimmune diseases, underlining their ability to induce ineffective and often inappropriate immunological responses [26].

**Fig. 3:**
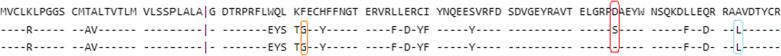
Alignment of HLA-DRB1*08:01 and HLA-DRB1*08:02 alleles using IPD-IMGT/HLA database

Indeed, Amoroso and his collaborators have been reported in a case–control Italian study that HLA-DRB1*08 was associated with almost doubled risk for Covid-19 in solid-organ transplant recipients and waitlisted candidates. In COVID-19 positive patients, HLA-DRB1*08 was correlated with mortality (6.9% in living versus 17.5% in deceased) [27]. Despite being from a population of transplant recipients and candidates, these results matched those of a case–control study conducted in the Midwest of the United States by Schindler et al. Where they found increased HLA-DRB1*08:02 in a small number of COVID-19 Hispanic patients. HLA-DRB1*08:01 is the dominant HLA-DRB1*08 allele found in Europe and is probably carried by most of the HLA-DRB1*08-positive cases in the Amoroso study. Of note, both HLA-DRB1*08:01 and HLA-DRB1*08:02 share the two risk-associated amino acid residues, Gly13 and Leu74, but differs in position 57 in Beta Helix domain, where HLA-DRB1*08:01 have the amino acid Ser while HLA-DRB1*08:02 have Asp at this position as shown in Fig. 3. The presence of aspartic acid at HLA-DRβ1 position 57 has been associated to leprosy risk [28], which could explain their low binding affinity for SARS-CoV-2 peptides [24]. This allele has been found to confer susceptibility to numerous auto-immune diseases such as Systemic Lupus Erythematosus [29]. Although these findings were limited to three observations among four Hispanics. Another study examining the frequency of HLA alleles among Saudi patients infected with COVID-19 found a significant increase in the frequency of HLA-DRB1*08 and also HLA-A*01, HLA-B*56 and HLA-DRB1*04 in the infected group compared to the control group, which remained significant after p-value correction [30]. Nonetheless, these results need further exploration using larger sample sizes from different regions and most likely, improved typing resolution.

In a study conducted in Spanish population, researchers found a tendency toward a higher rate of the allele HLA-A*32 in healthy controls than in COVID-19 patients, as well as the alleles HLA-B*39 and HLA-C*16 in COVID-19 patients than in healthy controls, on a total of 3886 healthy controls and 72 COVID-19 patients [31]. However, after accounting for multiple comparisons, the p-values remained non-significant. The lack of significant differences after Bonferroni correction might be explained by the limited sample size. Inversely, logistic regression analysis revealed that, after adjusting for SOFA or APACHE-II, the presence of HLA-A*11, HLA-C*01, and HLA-DQB1*04 alleles was related to higher mortality. These three HLA alleles associated with the mortality in this study, have previously been associated with poor evolution in other infectious diseases like HBV or active pulmonary tuberculosis (HLA-A*11 and HLA-DQB1*04) or with the risk of other infectious diseases such as acute viral encephalitis (HLA-C*01) [31]. As for Influenza A H1N1, A possible role of HLA-A*11 allele in conferring susceptibility has been found [32].

For the Japanese population, Anzurez et al*.*, analyzed HLA-A, HLA-C, HLA-B, and -DRB1 genotypes in 178 Japanese COVID-19 patients to investigate the association of HLA with severe COVID-19. This analysis revealed a significant association between HLA-DRB1*09:01 and COVID-19 severity. This HLA-Class II allele was more significantly associated with risk for severe COVID-19 in a cohort of Japanese patients compared to preexisting medical conditions. DRB1*09 frequency in study participants (14.2%) was found to be equivalent to that reported for the general Japanese population (14.5%), suggesting that this allele is not a risk-factor for SARS-CoV-2 infection. Thus, this association found in the present study may be attributed to skewed CD4+ T-cell responses in DRB1*09:01 positive patients. Further studies comparing SARS-CoV-2–specific CD4+ T-cells in infected individuals with and without the DRB1*09:01 allele are ongoing [33].

According to a Japanese study, the HLA-A*11:01:01:01 allele and the HLA-C*12:02:02:01-HLA-B*52:01:01:02 haplotype are significantly associated with the severity of COVID-19. The aim of this study was to spot susceptible HLA alleles and clinical markers that may be employed in risk prediction model for the early identification of severe COVID-19 among hospitalized COVID-19 patients. According to the Allele Frequency Net Database, HLA-C*12:02-HLA-B*52:01 haplotype is commonly present in Japanese with a frequency of 10.5%, followed by Asian populations residing in the USA (1.5–3.4%). On the other hand, HLA-A*11:01 is predominant in South-east Asia (17.7–61.3%), Japan (8.2–11.1%), China (16.2–61.3%) and the Oceania region (2.6–63.6%) [34]. Wang et al. in another publication reported the first host genetic investigation in the Chinese population by deeply sequencing and analyzing 332 COVID-19 patients with varied levels of severity, identified that the HLA-A*11:01, HLA-B*51:01, and HLA-C*14:02 alleles significantly predispose the worst outcome of the patients [35]. Accordingly, HLA-A*11:01 was significantly associated with both Japanese and Chinese severe COVID-19 patients, expecting that HLA-A*11:01 allele and HLA-C*12:02-HLA-B*52:01 haplotype could potentially act as a predictive marker for the severity of COVID-19 in the Asia region. It is remarkable that, in spite of the undeniable role of HLA and other host genetics. It is noteworthy that, certain HLA alleles can both offer protection and cause harm for the same disease, depending on the population and research design. As an example, Toyoshima et al., investigated the relationship between HLA-A*11:01 allele frequency and SARS-CoV-2 infection or mortality rate by comparing data from global databases and found that individuals with this allele could potentially be protected from SARS-CoV-2 infection, in contrast to study findings in this review [36].

Other studies, however, found no evidence of an association between SARS-COV-2 susceptibility or severity and HLA alleles in Spanish, Israeli, and South Asians populations [37–39].

These HLA molecules are expected to have a low affinity for SARS-COV-2 peptides, resulting in a dysregulated immune response that is unable to properly clear the virus and instead overreact, causing self-damage [22].

Regarding the differences among populations in that review, it should be accentuated that they vary significantly between areas, therefore it is quite feasible that alleles identified as risk alleles in certain association studies have little significance in other populations, due to their rarity in such population. However, some of these studies lack a sufficient number of cases to make a significant conclusion. This might explain the lack of consistency between studies.

These contradictory findings warrant further large, and reproducible studies to decipher any possible genetic predisposition underlying susceptibility to SARS-COV-2 and disease progression and host immune response. In this regard, many studies assessed the impact of polymorphisms in the genes that encode crucial renin-angiotensin system components which are ACE or ACE2 on the susceptibility, severity, and clinical outcomes of the COVID-19 infection, exploring the hypothesis that individuals with ACE polymorphisms may have a more severe disease course in response to infection with SARS-CoV-2 [40–44]. Again, findings are controversial. Some studies have not been able to show any association between ACE/ACE2 polymorphisms and COVID-19 clinical course, and the results lack validation.

Polymorphisms in ACE/ACE2 genes may change how SARS-CoV-2 binds or enters the body, causing more tissue damage in the lung and other organs [41].

Heterogeneous findings were observed regarding the correlation between genetic variations and COVID-19 susceptibility and severity [8, 9, 45–47].

Further investigation is required to clarify the functional mechanism by which genetic factors affect COVID-19 outcome and studies evaluating the mechanism of identified risk genes need to be conducted among populations with different ethnicities.

By identifying genetic markers that are associated with COVID-19 susceptibility or clinical outcome, a crucial contribution can be made to understanding this disease. This knowledge can then be used to or allowing us to detect those who are at risk, as well as to create strategies for vaccine and pharmacologic treatments.

## Conclusion

The interaction between the virus and the host immune system, in which the host HLA plays a critical role in the activation and modulation of the immune response, determines the clinical course of SARS-COV-2 infection. After reviewing and discussed all available data concerning the HLA polymorphism and susceptibility and/or severity of COVID-19 disease it seems likely the some HLA class I and class II alleles are associated to the COVID-19 disease. In fact, several alleles have been found associated with COVID-19 susceptibility and severity. Even results across articles have been inconsistent and, in some cases, conflicting, highlighting that the association between the HLA system and the COVID‐19 outcome might be ethnic‐dependent, there were some alleles in common between some populations such as HLA-DRB1*15 and HLA-A*30:02. In addition, HLA alleles as for example HLA-DRB1*08, HLA-DRB1*04, and others have been found to increase severity of the disease and are correlated with mortality. However, some of these articles lack a sufficient number of cases to make a significant conclusion. There is scope for further genetic diversity studies between populations on a larger scale to evaluate infection prognosis and vaccination efficacy. For the time being, these findings may furnish new perspectives into the pathological process of SARS-COV-2, the creation of novel antiviral molecules and the selection of therapies with greater immunogenic potential in order to reduce morbidity and mortality.

## Data Availability

All data generated or analyzed are included in this manuscript.

## Abbreviations

COVID-19: Coronavirus disease
SARS-CoV2: Severe acute respiratory syndrome 2
HLA: Human leukocyte antigen
MHC: Major histocompatibility complex
SARS: Severe acute respiratory syndrome
EBV: Epstein Barr virus
VZV: Varicella zoster virus
HHV7: Human herpesvirus 7
MCV: Merkel cell polyomavirus
ACE: Angiotensin-Converting Enzyme gene
